# 

**Published:** 1842-04-23

**Authors:** 


**CONTENTS OF No. 17.**

Dr. Gerhard on Diseases of the Heart and Aorta—with cases - - 257
Dr. Reynell Coates to the Editors - - - - - - - - 261
Dr. Reynell Coates' Contributions to Surgery:
Note on the means of avoiding Excoriations of the Heel and Perineum in Fractures of the Lower Extremities, treated by Permanent Extension - - 262
Dr. M'Pheeters' Case of Chronic Diarrhœa, treated by the internal use of the Nitrate of Silver - - - - - - - - - - - 264
BIBLIOGRAPHICAL NOTICES: Dr. Oliver Wendell Holmes on Homœpathy and its kindred Delusions - - - 265
A Circular Letter to the Physicians of Kentucky on Medical Reform - - - 266
EDITORIAL REMARKS.—On the Use of Injections of Rhatany in the Treatment of Fissure of the Anus, Prolapsus Ani, Leucorrhœa, &c. - 267
ANALECTA.—A new Remedy,—Clinkers, - - - - - - - 267
Professor Wawruch of Vienna on the Tape Worm - - 269
Dr. Hope on the Treatment of Acute Rheumatism - - 271
Dr. Hope on the Treatment of Chronic Rheumatism - - 272

